# Fluorescent Probes for STED Optical Nanoscopy

**DOI:** 10.3390/nano12010021

**Published:** 2021-12-22

**Authors:** Sejoo Jeong, Jerker Widengren, Jong-Chan Lee

**Affiliations:** 1 Department of New Biology, Daegu Gyeongbuk Institute of Science & Technology, Daegu 42988, Korea; sj960129@dgist.ac.kr; 2 Experimental Biomolecular Physics, Department of Applied Physics, Royal Institute of Technology (KTH), Stockholm 10691, Sweden; jwideng@kth.se; 3 New Biology Research Center, Daegu Gyeongbuk Institute of Science & Technology, Daegu 42988, Korea

**Keywords:** super-resolution microscopy, STED, fluorescent probe, fluorescent protein, organic dye, fluorescent nanoparticle

## Abstract

Progress in developing fluorescent probes, such as fluorescent proteins, organic dyes, and fluorescent nanoparticles, is inseparable from the advancement in optical fluorescence microscopy. Super-resolution microscopy, or optical nanoscopy, overcame the far-field optical resolution limit, known as Abbe’s diffraction limit, by taking advantage of the photophysical properties of fluorescent probes. Therefore, fluorescent probes for super-resolution microscopy should meet the new requirements in the probes’ photophysical and photochemical properties. STED optical nanoscopy achieves super-resolution by depleting excited fluorophores at the periphery of an excitation laser beam using a depletion beam with a hollow core. An ideal fluorescent probe for STED nanoscopy must meet specific photophysical and photochemical properties, including high photostability, depletability at the depletion wavelength, low adverse excitability, and biocompatibility. This review introduces the requirements of fluorescent probes for STED nanoscopy and discusses the recent progress in the development of fluorescent probes, such as fluorescent proteins, organic dyes, and fluorescent nanoparticles, for the STED nanoscopy. The strengths and the limitations of the fluorescent probes are analyzed in detail.

## 1. Introduction

An optical fluorescence microscopy is a powerful tool for studying microscopic objects in biological systems due to its ability to observe living cells, reasonable resolution (200–250 nm) for observing many subcellular features, noninvasive image acquisition, an option of multiplexed operation, and most importantly, chemical, and molecular specificity at single-molecule sensitivity. Sensitive and specific fluorescent labeling of biomolecules including proteins, nucleic acids, and lipids is indispensable for the success of modern fluorescence microscopy. Initially, fluorescent staining with molecular specificity was limited to several small molecules with intrinsic affinity to specific biomolecules and cell membrane permeability. A prominent example is the development of the DNA-specific stains DAPI [1] and Hoechst [2], which bind to the minor grooves of DNA and are now regarded as standards for nucleus staining in live cell imaging. Another good example is phalloidin [3,4] which binds to filamentous actin and label actin cytoskeletons in living cells. Small fluorescent molecules, also known as organic dyes or fluorophores, can be chemically linked to the stained molecules to generate or enhance fluorescence. Immunofluorescence expanded the viable targets of fluorescence labeling to essentially all proteins by using the highly specific binding of an antibody to its antigen [5,6,7]. Progress in antibody engineering has strongly enhanced the antibody–antigen specificity in immunofluorescence and has enabled the detection of single point mutations or post-translational modifications in proteins [8,9]. Antibodies, mostly secondary antibodies, must be chemically labeled with organic fluorophores to be used for immunofluorescence. Therefore, developing organic fluorophores with better photophysical and photochemical characteristics has been vital in improving immunofluorescence methodology. Nevertheless, immunofluorescence is typically not a method of choice for imaging living cells. Instead, target proteins can be labeled with fluorescent proteins (FP), such as GFP [10] and RFP [11], using genetic recombination facilitating specific and sensitive fluorescence labeling within living cells and organisms. After the nucleotide sequence of GFP was identified and cloned [12], recombinant GFP became one of the new standard tools for molecular biologists [13,14]. The progress was quickly followed by the development of fluorescent proteins with better characteristics such as enhanced fluorescence or accelerated protein folding [15,16].

Although the resolution in optical microscopy is limited by Abbe’s optical diffraction limit [17], a series of super-resolution microscopy or optical nanoscopy methods, such as PALM (Photo-Activated Localization Microscopy) [18], STORM (STochastic Optical Reconstruction Microscopy) [19], or STED (Stimulated Emission Depletion) nanoscopy [20], surpassed this limit using photophysical switching properties of fluorescent probes. The emergence of optical nanoscopy required fluorescent probes to have special photophysical and photochemical characteristics, and as a result, motivated the development of probes with novel characteristics. Conversely, optical nanoscopy could have only been experimentally demonstrated by the new types of probes that were then developed. For example, photoactivatable GFP, whose fluorescence could be turned ‘on’ via ultraviolet radiation, was the essential component in realizing PALM [18]. In the initial version of STORM, a Cy3–Cy5 dye pair was repurposed as a switch to achieve stochastic fluorescence emission [19]. In direct STORM (dSTORM), the photophysical property “blinking” or “fluorescence intermittency” is induced by particular redox buffers and utilized for super-resolution imaging, albeit it is usually avoided in most other fluorescence imaging tasks [21].

**Figure 1:**
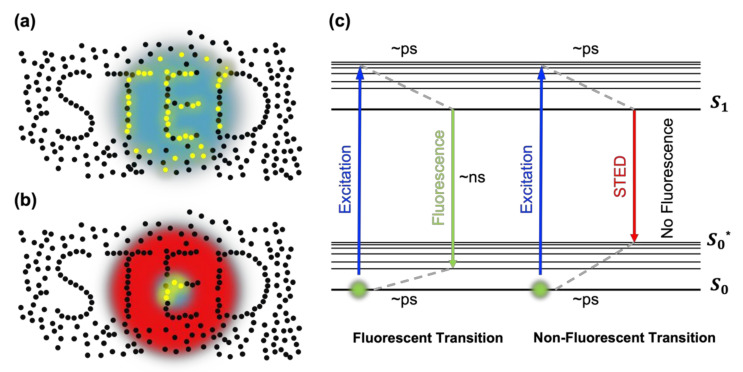
Basic working principle of STED nanoscopy. (**a**) Focal spot of conventional confocal microscopy. Fluorophores in the PSF of the excitation laser undergo fluorescent transitions. (**b**) Focal spot of the STED nanoscopy. Only fluorophores at the very center of the STED beam undergo fluorescent transitions. (**c**) Jablonski diagram of two molecular transitions: fluorescent transition (spontaneous emission) and nonfluorescent transition (stimulated emission).

In contrast to SMLM (Single-Molecule Localization Microscopy)-based approaches such as PALM, STORM, or dSTORM, STED optical nanoscopy takes a somewhat orthogonal approach rooted in point scanning confocal microscopy. When a fluorophore gets excited from the ground state to the excited state, it can release the absorbed energy as a fluorescent photon via spontaneous emission. Alternatively, it can release its energy via stimulated emission if there is a light beam carrying the same energy. It is, therefore, possible to force a fluorophore to cycle only in a transition involving stimulated emission but not in a transition involving fluorescence emission; this is called stimulated emission depletion (STED) [20]. In practice, STED nanoscopy achieves subdiffraction resolution by implementing a doughnut-shaped STED (or depletion) beam circumferent to a conventional Gaussian excitation beam (Figure 1a,b). At the center of the STED beam, ideally, the STED beam is absent, and only the excitation beam is present; hence, fluorophores are fully in the fluorescent cyclic transition as in conventional confocal microscopy (Figure 1c). In contrast, at the rim of the STED beam, where the intensity I is higher than a saturation (or threshold) intensity $I_{s}$, the stimulated emission outcompetes fluorescence emission, and the fluorophores are predominantly in the nonfluorescent cyclic transition (Figure 1c) [22]. The effective point spread function (PSF) becomes significantly smaller as the peripheral fluorescence is depleted by stimulated emission. The lateral spatial resolution of the STED optical nanoscopy becomes $\lambda \cdot {\left(2\mathrm{NA}\sqrt{1+I/I_{s}}\right)}^{-1}$ [22], where λ is the wavelength of the excitation beam and NA is the numerical aperture of the objective lens. This is a significant improvement over the diffraction-limited resolution of the conventional confocal microscopy $\lambda \cdot {\left(2\mathrm{NA}\right)}^{-1}$ [15].

An intrinsic advantage of STED nanoscopy over single-molecule localization microscopy is the higher temporal resolution since it does not require a large number of image frames and additional image processing [23]. A downside of STED nanoscopy, on the other hand, is the enhanced photobleaching of fluorophores as the intensity of the STED beam is typically 10^4^ to 10^5^ times stronger than that of the excitation beam. Decreasing the photobleaching in STED nanoscopy has been an important research direction [24,25]. Another important side effect of STED nanoscopy is the background noise due to the incomplete depletion and the unintended excitation by the STED beam [26]. Methods to suppress the background noise have been recently reported [26,27,28]. These problems in STED nanoscopy depend primarily on the photophysical and photochemical properties of fluorescent probes that are used. The properties of fluorescent probes are critical to the performance of the STED nanoscopy.

Developing fluorescent probes with certain photophysical and photochemical properties for the STED nanoscopy is essential. As the resolution of the STED nanoscopy is inversely proportional to $\sqrt{1+I/I_{s}}$, the probes should have low saturation intensity to minimize the STED beam dose. In other words, the probes need to be easily depletable at the STED wavelength. Additionally, the probes must be resistant to photobleaching, i.e., have high photostability [29]. The STED beam, introduced to deplete the fluorescent probes, can unintendedly or adversely excite the fluorophores. The fluorescent probes must thus also have low adverse excitability to minimize background noise. Furthermore, the fluorescent probes must be biocompatible or nontoxic to be used in live cell STED imaging. This review further discusses the requirements of fluorescent probes used in the STED optical nanoscopy in Section 2. An overview of recent progress in developing fluorescent probes, such as dedicated fluorescent proteins (FP), organic dyes, and fluorescent nanoparticles (FNPs), for STED nanoscopy, is presented in Section 3, along with the discussion of the strengths and limitations of these different fluorescent probes. Section 4 summarizes the review and offers a perspective on future outlooks.

## 2. Requirements for STED Probes

### 2.1. Photostability

**Figure 2:**
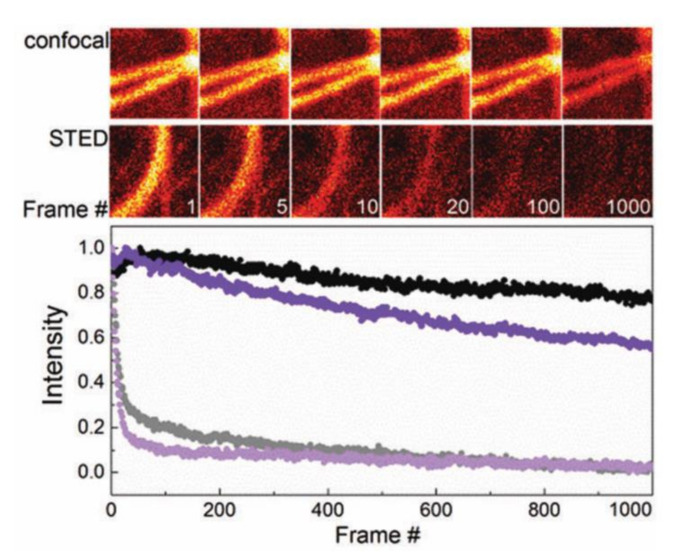
Top: Consecutive live cell fluorescence images of microtubules in COS-7 cells labeled with mGarnet2 using confocal microscopy and STED nanoscopy. Bottom: Normalized fluorescence intensity of the images as a function of the frame number upon confocal (black, violet) and STED (grey, light violet) imaging of live COS7 cells labeled with mGarnet (black, grey), and mGarnet2 (violet, light violet). Pixel dwell time: 40 μs. Excitation laser wavelength: 640 nm, Excitation laser power: 12.5 μW, STED laser wavelength: 780 nm, STED laser power: 36 mW. (Reproduced from Ref. [31] with permission from the Royal Society of Chemistry).

In STED optical nanoscopy, the donut-shaped STED beam is employed to deplete or de-excite the fluorescent probes at the periphery of the excitation beam (Figure 1). The depletion of the probes is related to the emission spectrum of the probes, the wavelength, and the power of the STED beam. Typically, a very strong STED power is required to achieve high resolution [30]. In conventional confocal microscopy, the excitation beam typically has a few μW of power; hence, most commercial probes can be used without photobleaching problems. In STED nanoscopy, however, the power of the STED beam can be tens to hundreds of mW. Consequently, fluorescent probes in STED nanoscopy are subject to stronger photobleaching than in confocal microscopy (Figure 2) [31]. Figure 2 shows the repeated image acquisition of microtubules in COS-7 cells labeled with the fluorescent proteins mGarnet and mGarnet2. They retained most of their fluorescence over around 1000 image acquisitions when acquired by confocal microscopy. However, the same samples were significantly photobleached after 20 image acquisitions with STED nanoscopy. This illustrates that fluorescent probes should have high photostability to be used in STED nanoscopy.

### 2.2. Depletability

In STED nanoscopy, the fluorescent probe must be efficiently depleted by the STED beam to achieve diffraction-unlimited resolution. Depletability of a fluorescent probe here refers to its tendency to be depleted by a STED beam of choice. Depletability can be quantified by calculating the rate equations of electronic transitions in the STED process. In general, any light-driven transition rate from an electronic state (a) to another electronic state (b) can be denoted as: $k_{ab}=\underset{i}{\sum }\sigma_{ab}\left(\lambda_{i}\right)\cdot I_{i}\left(\lambda_{i}\right)$, where $\sigma_{ab}\left(\lambda_{i}\right)$ is the cross section of the transition $\lambda_{i}$ is the wavelength of light, and $I_{i}\left(\lambda_{i}\right)$ is the intensity of the laser beam at $\lambda_{i}$ [32]. Then, depletability can be written as: $k_{\mathrm{STED}\;S10^{*}}=\sigma_{S10^{*}}\left(\lambda_{\mathrm{STED}}\right)\cdot I_{\mathrm{STED}}$, where $k_{\mathrm{STED}\;S10^{*}}$ represents the transition rate from S1 to $S0^{*}$ induced by the STED beam, $\sigma_{S10^{*}}\left(\lambda_{\mathrm{STED}}\right)$ is the cross section of the transition S1 to $S0^{*}$ at $\lambda_{\mathrm{STED}}$, and $I_{\mathrm{STED}}$ is the intensity of the STED beam. Here, to enhance depletion efficiency, increasing the stimulated emission cross section $\sigma_{S10^{*}}\left(\lambda_{\mathrm{STED}}\right)$ is often necessary. The stimulated emission cross section has a spectral dependency, $\sigma_{S10^{*}}\left(\lambda \right)=\lambda^{4}E\left(\lambda \right)\phi_{f}/\left(8\pi cn^{2}\tau \right)$ [33], where λ is the wavelength, $E\left(\lambda \right)$ is the normalized emission spectrum at wavelength λ, $\phi_{f}$ is the fluorescence quantum yield of the probe, c is the speed of light, n is the refractive index, and τ is the excited state lifetime. The wavelength of the STED laser must be overlapped with the red tail of the emission spectrum $E\left(\lambda \right)$ to achieve reasonable stimulated emission cross section. *Vicidomini* et al., and *Bordenave* et al., reported that changing the STED wavelength $\lambda_{\mathrm{STED}}$ to the probe’s emission maximum makes the stimulated emission cross section ($\sigma_{S10^{*}}$) 10-fold larger than that at the red tail of the emission spectrum [34,35]. Using a STED wavelength in the middle of the emission spectrum, however, results in a significant scattering noise that is undetachable with the fluorescence signal. It is, therefore, impractical to blue-shift the STED wavelength for the sake of increasing stimulated emission cross section. A proper probe with a reasonably long and strong red tail in the emission spectrum is required.

Generally, quantum dots (Qdots) have longer and stronger red emission tails compared with organic fluorophores or fluorescent proteins. For example, ZnS-coated CdSe Qdot705 has high emission intensity at the 775 nm depletion laser wavelength, 18% of its emission maximum, [27] while other organic dyes or fluorescent proteins, such as Atto 647N, AberriorStar 635, or mGarnet2, show 5%, 7% and 10% of their emission maximum, respectively. Although there exist other factors that affect the stimulated emission cross section, such as the fluorescence quantum yield $\phi_{f}$, and the excited state lifetime τ, their contribution is less dominant than the emission intensity [31,36,37]. Therefore, Qdots, in most cases, have higher depletability compared with organic fluorophores or fluorescent proteins.

A high stimulated emission cross section value ($\sigma_{S10^{*}}$) reduces the requirements on the saturation intensity $I_{s}$ [24]. The saturation intensity can be written as: $I_{s}=k_{\mathrm{exc}\;s10}hc/\left(\lambda_{STED}\sigma_{S10^{*}}\right)$, where $k_{\mathrm{exc}\;s10}$ is the spontaneous decay rate, and $hc=1.99\times 10^{-25}$ Jm. A higher $\sigma_{S10^{*}}$ lowers the saturation intensity $I_{s}$; therefore, the same super-resolution is achieved at a lower STED beam intensity. High stimulated emission cross section results in multiple benefits. By lowering the STED beam intensity, the photodamage of the sample can be reduced. Also, the photobleaching of the fluorescent probe is reduced. Another benefit of having a high $\sigma_{S10^{*}}$ is that the signal-to-noise ratios (SNRs) in the images can be enhanced. There are two significant kinds of background noise specifically appearing in STED nanoscopy: incomplete depletion noise and direct excitation noise [32]. Incomplete depletion noise takes place primarily at the periphery of the doughnut-shaped STED beam, where the STED intensity is below the saturation intensity. At the periphery, the fluorescence cannot be fully suppressed, and the resulting fluorescence leakage contributes to the background noise. If the probe has a low saturation intensity and the peripheral area where the STED intensity falls below the saturation, the intensity would be reduced, and the incomplete depletion noise can be suppressed. Furthermore, reducing STED beam intensity due to lower saturation intensity lowers the direct excitation noise retaining the same resolution. Direct excitation noise is discussed in the following subsection in detail.

### 2.3. Adverse Excitability

Even if the probe has a high depletion efficiency, other probe properties can compromise the STED image quality. Ideally, the STED beam functions as a fluorescence suppressor such that it stimulates the decay from $S_{1}$ to $S_{0}^{*}$. However, there is a probability that the STED beam unintendedly or adversely re-excites the molecule from $S_{0}^{*}$ to $S_{1}$ or directly excite it from $S_{0}$ to $S_{1}$. This anti-Stokes re-excitation generates a background noise profile, named the direct excitation noise [24]. Since the emission spectrum of a probe is red-shifted compared to that of absorption or excitation (Figure 1c), the STED beam wavelength is typically chosen such that overlap with the absorption spectrum is minimized while retaining the cross section of the stimulated emission ($\sigma_{S10^{*}}$) reasonably high. By doing so, the cross section of direct excitation $\sigma_{S0^{*}1}\left(\lambda_{\mathrm{STED}}\right)$ becomes small, and unintended anti-Stokes re-excitation can then be neglected. On the other hand, when $\sigma_{S0^{*}1}\left(\lambda_{\mathrm{STED}}\right)$ is not negligible, the anti-Stokes re-excitation causes a disturbing background problem. Hence, a probe must have small adverse excitability, i.e., a small excitation cross-section in the red tail of the emission spectrum, to be used for high-quality STED imaging [38]. A probe with a large Stokes shift between excitation and the emission spectrum is favorable [39].

Several technical efforts have been made to suppress the STED-specific noise sources [26,27,28,40]. For example, polarization switching STED (psSTED) inhibits both incomplete depletion noise and direct excitation noise by switching polarization of STED light beam [26]. Switching the polarization from one circular polarization to another, the hollow STED beam changes into a center-filled STED beam with which only noise is collected. The psSTED gathers a regular STED image with one polarization and a noise image with a perpendicular polarization; then, a noise suppressed image can be obtained by subtracting the two. Nevertheless, technically suppressing STED-specific background noise has several limitations. Most of the techniques require either multiple scans or the installation of extra equipment such as time-correlated single-photon counting (TCSPC). Multiple scans can induce additional photobleaching. Adding an extra device is challenging for commercial STED microscopes and often costly. It is, therefore, beneficial to use a probe that has small adverse excitability and good depletability.

Generally, Qdots have a long red tail in the excitation spectrum and a high overlap of excitation spectrum and stimulated emission wavelength compared with organic dyes and fluorescent proteins. Qdots have high adverse excitability, although they have good depletability [27]. As a result, Qdots exhibit strong direct excitation noise while their incomplete depletion noise is marginal. For instance, with high STED beam power (>200 mW), the anti-Stokes adverse excitation noise can be comparable to the fluorescence signal itself, about 30% or more of the maximum fluorescence intensity [41]. It is, therefore, challenging to use Qdots as probes for STED nanoscopy typically. A method to suppress adverse excitation noise from Qdots using double scans has been demonstrated [27]. In the two consecutive scans, the first scan is a regular STED imaging that contains severe noise, and the second scan is performed only with the STED beam. The second scan image records only noise information and subtracting the second image from the first image produces a noise subtracted image.

### 2.4. Biocompatibility

**Figure 3:**
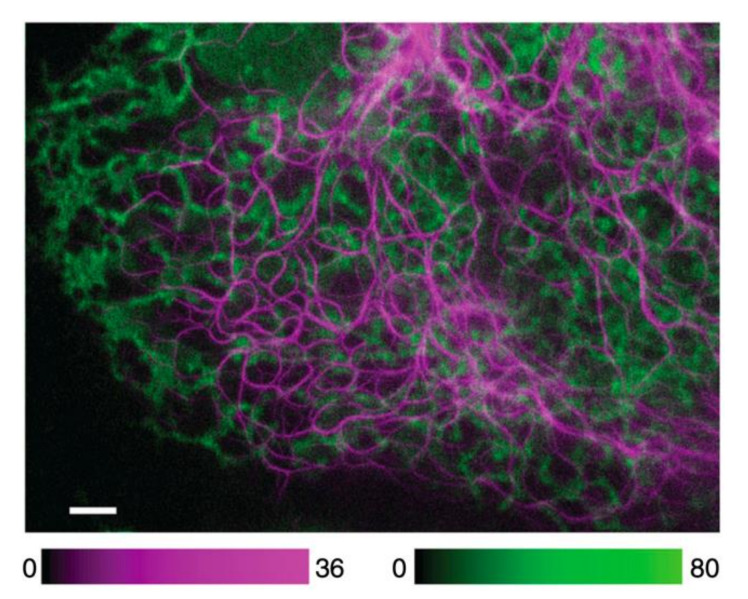
Red confocal/NIR STED dual-color imaging. VIM-SNIFP (magenta) was recorded in STED mode, and the ER targeted mCherry (green) in confocal mode. Scale Bar: 2 μm. Material drawn from [45].

One of the reasons to use optical nanoscopy is the live cell imaging capability. During imaging living cells in time series, the cells must be maintained in healthy status. Therefore, as a first criterion, the probes in use must be nontoxic or minimally toxic to the cell. Generally, nonmetal probes are less cytotoxic for biological samples, while heavy-metal-cored nanoparticles are cytotoxic. Intracellular oligomerization or aggregation of probes can also cause cytotoxicity, although they are not cytotoxic as a monomer. For example, some FPs tend to form potentially cytotoxic oligomers in cytosols and lysosomes [42,43]. In STED nanoscopy, with the use of a strong STED beam, phototoxicity becomes a significant issue, limiting the selection of probes. The use of plasmonic nanoparticles in STED nanoscopy can generate photothermal effects around the probe, which might damage a living cell [44]. Strong light irradiation leads to the formation of free radicals and singlet oxygens, which are further reinforced by fluorescent probes in the sample. The effects are often nonlinear and thus depend on how the excitation light dose is distributed in time onto the sample [32]. Irradiating light wavelength is closely related to phototoxicity; therefore, it is often a good strategy to use red-shifted wavelengths for excitation and depletion [45]. In the near-infrared (NIR) range, around 650–900 nm, phototoxic phenomena are typically much lower than in the visible range at high light doses. Furthermore, scattering, autofluorescence, and absorbance are remarkably reduced within the NIR window for mammalian cells and tissues [31]. In this respect, probes working in the NIR range have higher biocompatibility compared with those working in the visible range. Also, developing probes in the NIR range can extend the possibilities for multicolor STED imaging (Figure 3) [45].

It is also imperative for the probes to have ease of labeling and high target specificity. Immunofluorescence is the most widely used methodology for fluorescence microscopy [46,47,48]. The antibodies used for immunofluorescence are often quite large; for instance, a typical IgG antibody is ~150–170 kDa. As a result, fluorescently labeled antibodies require enhanced permeability through the cellular membranes, and they often tend to aggregate in the cell, which can cause cytotoxicity and imaging artifacts. In addition, as the resolution in super-resolution microscopy approaches a few tens of nanometers or better, the size of primary–secondary antibody heterodimer can disturb accurate position assessment of the target molecule. One interesting alternative to antibodies is the use of affinity molecules of smaller size, such as aptamers, affibodies, and nanobodies [32]. Active targeting can be another alternative to immunofluorescence [49,50]. By controlling the hydrophobicity or hydrophilicity of the probe, surface probes can be preferentially targeted to, for instance, mitochondria or nucleic acids [51]. Many specific targeting methods can thus be suggested for live cell imaging under harsh light irradiation conditions [52,53,54]. It is worth noting that FPs genetically fused to the target molecule tend to have high target specificity as well as ease of labeling. In the case of organic dyes, the development of labeling methods utilizing fusion tags made the labeling accessibility and target specificity comparable to those of FPs [55].

## 3. Progress in Development of Fluorescent Probes for STED Nanoscopy

### 3.1. Fluorescent Proteins (FPs)

The discovery and utilization of FPs have made targeted fluorescence labeling much more convenient and have generated significant growth in the field of fluorescence microscopy [10]. The most powerful and convenient aspect of FPs is that they can be genetically included into, and expressed by, living cells without any externally added chemicals. However, due to their lower brightness and photostability compared with organic dyes and fluorescent nanoparticles, FPs are not widely used in STED nanoscopy [56,57,58,59]. Nevertheless, there has been progress in the development of FPs in the far-red to NIR wavelength range which have higher photostability and improved fluorescence brightness, such as TagRFP657, mNeptune2, mGarnet and mGarnet2 [31,45,60,61,62,63]. These NIR FPs also benefit from lower phototoxicity and absorption thanks to their operating wavelengths, as discussed above [31,45]. Their NIR emission spectrum allows the STED beam wavelength to be further red-shifted in the NIR optical window, but many require a shorter excitation beam at the red or far-red spectrum. Phytochromes excitable at the NIR spectrum have been developed by further red-shifting the phytochromes’ spectra [45]. Both excitation and emission at the NIR spectrum are favorable due to low phototoxicity, low absorption, and reduced overlap with red probes in multicolor red-confocal/NIR STED imaging [45] (Figure 3). Still, mainly FPs exhibit strong photobleaching by the intense STED beam (Figure 2), which limits the use of FPs in prolonged STED imaging [45].

Fluorescent proteins are more often adopted in another super-resolution microscopy technique RESOLFT (REversible Saturable OpticaL Fluorescence Transitions), which share a similar working principle with STED nanoscopy [64]. In STED nanoscopy, the STED doughnut beam de-excites the probe into a ground state by stimulated emission. Similarly, in RESOLFT, a doughnut-shaped ‘off’ beam is used to switch probes into a dark ‘off’ state where the probes are unable to emit fluorescence photons even if they are illuminated with the excitation beam. As a result, probes at the center of the doughnut beam remain in a bright ‘on’ state while the periphery probes are in a dark ‘off’ state, making the subdiffraction imaging possible. The dark “off” states exploited in RESOLFT can be induced with much lower light intensities. Therefore, unlike STED nanoscopy, which typically requires tens of mW of STED laser power, RESOLFT can use illumination power that is a few orders of magnitude lower [64]. This relieves the requirements for photostability and brightness for FPs compared to FPs for STED applications. However, to selectively turn the probes ‘on’ and ‘off’, special kinds of FPs, so-called photoswitchable FPs (PS-FPs), are necessary [65,66,67,68,69]. Hofmann et al. demonstrated the RESOLFT concept by using a reversibly photoswitchable FP, asFP595, activated at 568 nm and turned ‘off’ at 458 nm [64]. Andresen et al., and Dedecker et al., used Dronpa as the PS-FP, whose brightness is twice that of EGFP or higher [65,70,71]. The high fluorescence quantum yield of Dronpa was favorable for live cell protein tracking experiments [70,72,73]. An important limitation of PS-FPs is their slow switching kinetics, limiting imaging speed and brightness. Therefore, faster switching PS-FPs for RESOLFT have been developed [74,75]. A PS-FP with higher switching efficiency, thus with higher brightness, is demonstrated [76]. RESOLFT, with its ability to manipulate fluorescence using a much lower light dose than STED, can be the method of choice in prolonged super-resolution imaging. In vivo multicolor RESOLFT super-resolution imaging of mouse cortex using three different labels, EGFP, Citrine, and rsEGFP2, has been demonstrated [77]. As an additional strategy to increase imaging speed and take advantage of the relatively low laser intensity requirement, RESOLFT can also be implemented with highly parallelized doughnut beams [78].

There is still room for further development of FPs and PS-FPs for STED and RESOLFT optical nanoscopy. Highly photostable and bright NIR FPs are desirable for STED applications. In the case of RESOLFT, high brightness and fast switching are the favorable properties of PS-FPs to be developed.

### 3.2. Organic Dyes

Organic dyes are most commonly used in STED nanoscopy because they are relatively photostable under the relatively demanding light irradiation conditions and are generally much brighter than FPs [79,80,81,82,83]. Among the organic dyes, rhodamine-based Atto647N, which has high fluorescence quantum yield and large extinction cross section, has been widely used as a probe for STED nanoscopy [84,85]. KK114 is another successful option for STED imaging based on a Rhodamine [86,87,88,89]. However, the use of KK114 is limited due to its low cell permeability and low NHS-ester stability. To overcome these limits, Wurm et al. developed new rhodamine-based red fluorophores, KK1119, KK9046, Abberior STAR 635, and Abberior STAR 635P, which exhibit higher quantum yields and improved NHS-ester stabilities [90]. Fluorescent rhodamines and fluorogenic carbopyronines with better cell permeability were also developed [91]. Organic dyes become much more useful when combined with fusion tags such as HaloTag and SNAPTag [55]. A target protein can be genetically fused with a peptide tag and the fusion protein expressed in the cells; the tagged target protein can then be labeled with ligand-modified fluorophores added to the cells.

STED optical nanoscopy is often carried out in multiple colors, which requires the careful selection of organic dyes. In a conventional multicolor STED imaging design, two different dyes could share a single depletion beam wavelength. In doing so, the instrumentation complexity is lowered, the detection volumes of the two emission bands are automatically overlaid by a common STED beam, and suppression of elastic and Raman scattering into the fluorescence bands from multiple lasers becomes less of a concern. However, spectral crosstalk between two dyes can still occur and can affect the image quality. By alternating pulsed excitation or so-called pulsed-interleaved excitation, such crosstalk can be kept low [92]. It is, for example, possible to find a commercial STED nanoscopy instrument carrying alternating 595 nm and 640 nm excitation pulsed lasers, together with a 775 nm laser for depletion. Nevertheless, for efficient multicolor STED imaging, probes whose emission spectra are further blue shifted, compared to red, far-red, or orange-emitting dyes, can sometimes be useful as second or third labels depletable with an additional STED beam. Such STED nanoscopy instruments are commercially available, for example, with two STED wavelengths located at 592 nm (or 660 nm) and 775 nm. Currently, there are many commercial organic dyes available for STED imaging with depletion beams at 592, 660 and 775 nm, such as OG488, AF555 and SiR. Still, there are efforts to develop organic dyes with better photophysical and photochemical characteristics for STED nanoscopy. For example, Grimm et al. developed green-emitting rhodamine-based organic dyes depletable at 595 nm and suitable for live cell imaging [93].

**Figure 4:**
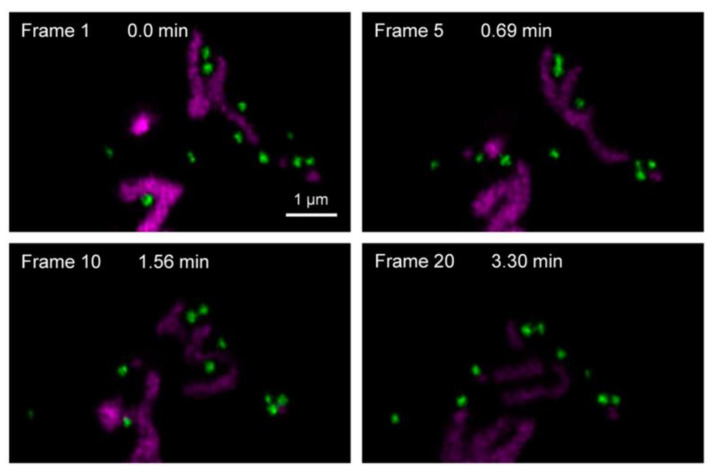
Two-color STED super-resolution imaging of living HeLa cells. The lipid droplets (green) and mitochondria (purple) were labeled with Lipi-DSB and TMRM, respectively (scale bar = 1 μm); the STED images were recorded under two excitation lasers (470 nm for Lipi-DSB, 550 nm for TMRM) and one STED laser (660 nm CW-STED, 40 MW cm^−2^). Reprinted (adapted) with permission from Zhou, R.; Wang, C.; Liang, X.; Liu, F.; Yan, X.; Liu, X.; Sun, P.; Zhang, H.; Wang, Y.; Lu, G. Stimulated Emission Depletion (STED) Super-Resolution Imaging with an Advanced Organic Fluorescent Probe: Visualizing the Cellular Lipid Droplets at the Unprecedented Nanoscale Resolution. ACS Mater. Lett. 2021, 3, 516–524, Copyright 2021 American Chemical Society.

As mentioned in Section 2.3., the performance of STED nanoscopy can sometimes be improved if the probe has a significant Stokes shift. Following this strategy, Zhou et al. recently developed a distyrylbenzene-based Lipi-DSB fluorophore that stains lipid droplets (Figure 4) [94]. Lipi-DSB shows a large Stokes shift, low saturation intensity, and high photostability. In Figure 4, Lipi-DSB remains in a bright fluorescent state, while mitochondria stained with TMRM (Tetramethyl rhodamine methyl ester) are bleached out after 20 frames of STED illumination. O’Connor et al. also developed a pyrene-based ceramide conjugate PyLa-C17Cer probe with a large Stokes shift and stains LD (Lipid Droplet) [95]. Thanks to its high target specificity, low cytotoxicity, and high photostability compared to PyLa, PyLa-C17Cer is more suitable for live cell imaging. Yang et al. used a squaraine-based probe MitoESq-635 that exhibits high photostability and low saturation intensity, thus, enables long-term live cell imaging [96]. Mitochondrial inner membranes were imaged over 50 min in a living Hela cell with a resolution of 35.2 nm.

**Figure 5:**
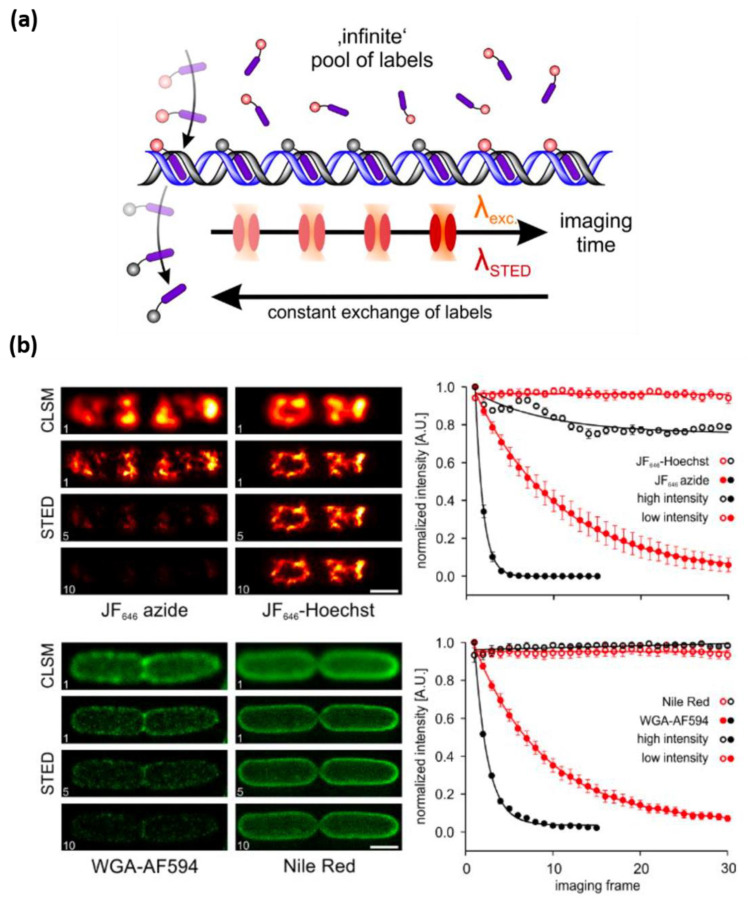
STED nanoscopy with exchanging probes. (**a**) Fluorophores that dynamically bind and unbind from their target structure ensure a constant exchange and allow long-term STED imaging. (**b**) Repeatedly acquired STED nanoscopy images using regular and reversibly binding labels targeting the *E. coli* membrane or nucleoid. Cells were labeled with immobile (left images) or exchanging probes (right images). A high concentration of the exchangeable dyes JF_646_-Hoechst and Nile Red was applied for the dynamic labeling of DNA (red) and *E. coli* membranes (green), respectively. Graphs on the right show intensity time trace of regular STED image, using exchanging probes at high and low STED laser intensities. Reprinted (adapted) with permission from [97].

To overcome photobleaching in STED nanoscopy, the exchangeable fluorophore system could be exploited, which prevents photobleaching by constantly replacing photobleached probes with new ones [97]. It has some similarities to the PAINT (Points Accumulation for Imaging in Nanoscale Topography) imaging method, which is based on the single-molecule localization [98]. In an exchangeable fluorophore system (Figure 5a), many probes are floating in the medium undetected because their fluorescence signals are averaged out as a background. When the probe is attached to the target molecule, the motion of the probe is restrained, and the fluorescence signal is detected. Probes floating in a medium repetitively attach and detach to the target, replenishing unbleached fluorophores. As a result, the photobleaching at the target molecule becomes negligible. Figure 5b shows that this strategy efficiently suppressed photobleaching in repeated STED imaging. If the photobleaching can be reduced, either by a new probe or a clever bypass, STED intensity can be increased, and the spatial resolution of STED nanoscopy can be improved.

### 3.3. Fluorescent Nanoparticle (FNP)

It is well known that photobleaching is significantly reduced when organic dyes are embedded in a rigid environment [99]. If a nanoparticle is decorated with fluorescent molecules tightly aligned or aggregated on its surface, it can function as a bright and photostable fluorescent nanoprobe and can be used for STED imaging [100]. These kinds of heavily dye-loaded nanoprobes are called AIE NPs (aggregation-induced emission nanoparticles) because they are synthesized using a synthetic dye that shows aggregation-induced emission (AIE) [101,102,103,104]. After functionalizing the surface with streptavidin, the AIE NP was shown to work well as a STED probe in imaging subcellular structures of fixed MCF-7 cells [105]. However, poor biocompatibility limits the applicability of AIE NPs.

**Figure 6:**
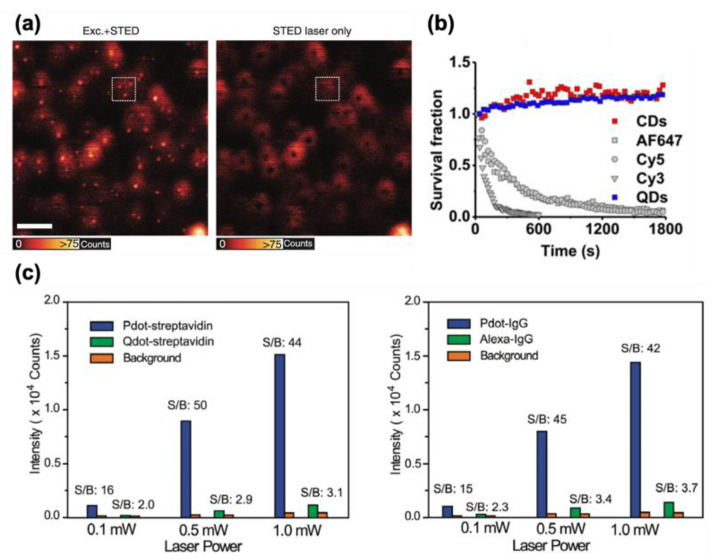
(**a**) Left: Qdot705 imaged with STED nanoscopy, i.e., with both the STED and the excitation beam. Right: The same sample imaged only with the doughnut-shaped STED beam. (**b**) Survival fraction during continuous STED illumination compared for different types of fluorescence probes. (**c**) Left: Average fluorescence brightness and SNR of Pdot−streptavidin and Qdot−streptavidin. Right: Average fluorescence brightness and SNR of Pdot−IgG and Alexa 488−IgG. (**a**) Reprinted (adapted) with permission from [27]. Creative Commons Attribution 4.0 International License. (**b**) Reprinted (adapted) with permission from [106]. Copyright 2021 American Chemical Society. (**c**) Reprinted (adapted) with permission from [111]. Copyright 2021 American Chemical Society.

Inorganic semiconductor quantum dots (Qdots) have the potential as photostable and bright probes for STED nanoscopy. However, typically Qdots exhibit severe direct excitation background noise, which must be suppressed for high-quality STED. To solve the background noise problem, samples can be scanned once again only with the STED laser on to record direct excitation noise image, and then the noise image can be subtracted from the original STED image (Figure 6a) [27]. Despite its poor SNR and cytotoxicity, Qdots remain attractive probes for STED imaging because they are extremely bright and highly resistant to photobleaching (Figure 6b, blue dot) [106]. To avoid the anti-Stokes re-excitation background noise, Ye et al. used CdSe@ZnS Qdots for STED imaging [107]. They found that CdSe@ZnS Qdots barely emit direct excitation noise within the green emission range. With an excitation laser at 488 nm and STED laser at 592 nm, 21 nm of the resolution was accomplished without much noise. Recently, halide perovskite ($\mathrm{CsPb}X_{3},$ X=Cl, Br, or I) Qdots have received attention for their good optical properties as STED probes and low synthesis cost [108,109]. ${\mathrm{CsPb}\mathrm{Br}}_{3}$ Qdots show extremely high photostability and low saturation intensity while exhibiting 20.6 nm resolution under 39.8 mW of STED laser for 200 consecutive minutes. Nevertheless, Qdots tend to have low biocompatibility and high cytotoxicity. Carbon dots (Cdots) have also been considered as probes for bioimaging because of their biocompatibility, high photostability, tunable emission spectra, and high quantum yields [29]. By virtue of their optical properties, Cdots have found various applications in the fields of bioimaging, both for in vitro and in vivo research. Figure 6b shows that Cdots can be as photostable as Qdots and thus could be used for long-term live cell imaging. Fluorescent semiconductor polymer dots (Pdots) can also be used as STED probes thanks to their high brightness, photostability, and low cellular toxicity. Wu et al. adopted Pdots for STED imaging and demonstrated high biocompatibility, photostability, and good depletability [110]. Pdots could be conjugated with molecules such as biotin and showed a very low STED power requirement (<3 mW for 70 nm resolution). It is demonstrated that Pdots remain fluorescent after two hours of live cell imaging. Another report pointed out that Pdots have higher SNR than Qdots or organic dyes (Figure 6c) [111]. On the other hand, there are weaknesses of FNPs due to their relatively large size. It is typically difficult to use FNPs for intracellular labeling because FNPs tend to have compromised membrane permeability and large steric hindrance.

## 4. Conclusions and Outlook

Progress in STED optical nanoscopy was inseparable from the development of fluorescent probes for STED. In some cases, new methodologies for STED nanoscopy were developed to overcome the limitation of the available probes. On the other hand, probes with better photophysical and photochemical characteristics for STED nanoscopy were developed. This review discussed the requirements of probes to be suitable for STED nanoscopy. A proper STED nanoscopy dye must meet four major requirements: high photostability, good depletability, low adverse excitability, and good biocompatibility. These requirements are closely related to the nature of STED nanoscopy and the use of a strong STED beam. The methods that can circumvent the background noise problem arising from low depletability or high adverse excitability have been developed. Although there are technical measures to ease the probe requirements, this review showed that it is essential to have probes with good properties in the first place. Different kinds of probes that have been developed and implemented in STED nanoscopy so far have been identified and discussed. It was noted that fluorescent proteins were developed for STED nanoscopy but were not heavily used due to their relatively low photostability and brightness. Nevertheless, they have been actively developed for RESOLFT nanoscopy, which shares a similar working principle with STED nanoscopy. The review also discussed the development of organic dyes as the most widely used fluorescent probes for STED nanoscopy. Several developments to enhance the brightness, photostability, and biocompatibility of organic dyes have been reported. Adjusting the probe’s excitation and emission spectrum was an important research direction for better multicolor STED imaging. Lastly, different kinds of fluorescent nanoparticles have been introduced. AIE NPs were bright and highly photostable but have compromised biocompatibility. Qdots were bright and photostable but had strong adverse excitation. Methods to overcome the noise originating from Qdots’ adverse excitation were also discussed. Qdots tend to have high cytotoxicity, similar to AIE NP. We introduced recently developed Cdots and Pdots, which are biocompatible, photostable under intense STED laser, and easily depletable, making them potentially suitable STED probes.

Interestingly, no single fluorescent probe meets all the requirements simultaneously for the STED nanoscopy. Therefore, there seems to be ample room for further development of FPs, organic dyes, and FNP and the development of STED techniques to compensate for the probes’ weaknesses and achieve high-quality STED imaging.
